# Logic model development and use in geriatric education programs

**DOI:** 10.1093/geroni/igaf122.1373

**Published:** 2025-12-31

**Authors:** Rhiannon Williams

**Affiliations:** University of Minnesota, Minneapolis, Minnesota, United States

## Abstract

This presentation will provide a brief overview of what a logic model is and how it can be used to support the development, implementation, and assessment of one’s geriatric education program/ projects. A logic model is a picture or visual representation of how a program does its work – the theory and assumptions underlying the program, the activities it undertakes, and how those actions are thought to lead to desired results/outcomes. For those that design and use a logic model, it provides a shared understanding around an initiative/program, is flexible, and can be updated, as well as serve as a framework for evaluation: one can evaluate any of the logic model steps or the linkages between steps. The presentation will provide attendees with the components of a logic model, a description of each component; inputs/resources, activities, products/outputs, short-term outcomes, and long-term outcomes, using the case of the interprofessional geriatric case competition. We will give tips on how to create and use one in the development and checking of logic of a program. Finally, the presentation will explain how subsequent presentations provide examples of evaluation at different points along the logic model. Attendees will have a clear understanding of the value of a logic model and how they could use one with their geriatric education program.

